# Computational Predictive Modeling of Surgical Outcomes in Total Anomalous Pulmonary Venous Connection: Assessing the Impact of Pulmonary Venous Confluence Size on Preoperative Planning

**DOI:** 10.1007/s12265-025-10725-9

**Published:** 2026-02-04

**Authors:** Jie Jin, Zhuo Shi, Qiang Gao, Jing Yu, Irina Jin, Jiawei Liang, Xiangming Fan

**Affiliations:** 1 Department of Cardiac Surgery, The Children’s Hospital, Zhejiang University School of Medicine, #3333, Binsheng Road, Binjiang District, Hangzhou, 310052 China; 2 Department of Radiology, The Children’s Hospital, Zhejiang University School of Medicine, Hangzhou, China; 3 Department of Ultrasonography, The Children’s Hospital, Zhejiang University School of Medicine, Hangzhou, China; 4 National Center for Materials Study and Testing, Technical University of Moldova, Stefan Cel Mare Av. 168, MD-2004 Chisinau, Republic of Moldova

**Keywords:** TAPVC, Fluid–structure interaction, Equation, Pulmonary vein, Anastomosis

## Abstract

To develop a predictive model for optimal anastomosis sizing in TAPVC surgery, focusing on the role of pulmonary venous confluence (PVC) size. A patient-specific fluid–structure interaction (FSI) model simulated blood flow through various anastomosis sizes. Key variables included body weight, anastomosis length, and PVC size. The model's predictions were validated against postoperative echocardiographic measurements from nine TAPVC cases. A strong positive correlation was found between flow velocity and the ratio of body weight to anastomosis length and PVC circumference. Including PVC size significantly improved predictive accuracy. No significant difference was observed between predicted and measured velocities. PVC size is a critical factor for planning TAPVC surgery. Incorporating it into computational models enhances the prediction of flow dynamics and supports personalized surgical decision-making.

**Graphical Abstract:**

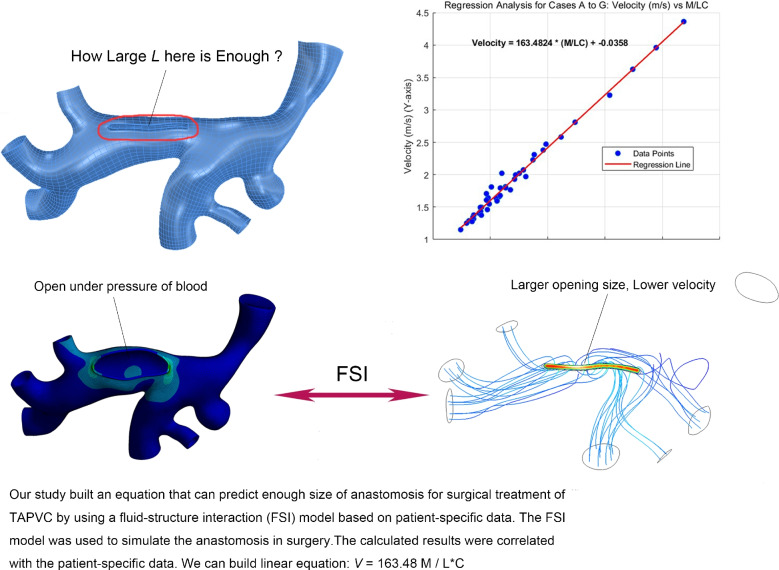

**Supplementary Information:**

The online version contains supplementary material available at 10.1007/s12265-025-10725-9.

## Introduction

Total anomalous pulmonary venous connection (TAPVC) is a complex and rare cardiac malformation in which the pulmonary veins abnormally connect to systemic veins or the right atrium instead of the left atrium [1]. Despite significant advancements in surgical techniques and pre/postoperative care over the past decades, correcting TAPVC remains a challenging task. A major complication and leading cause of reoperation in TAPVC patients is postoperative pulmonary venous obstruction (PVO) [2, 3], which significantly impacts long-term survival and quality of life. The recurrence of PVO is associated with factors such as endothelial fibroblast proliferation, primary pulmonary vein stenosis [4], and the size of the anastomosis created during surgery. Despite advances in surgical techniques, the incidence of postoperative PVO remains approximately 10–20% in clinical studies [5, 6], and Doppler-derived flow velocity across the anastomosis is widely applied as a clinical indicator, although the exact velocity thresholds vary across reports. To prevent PVO, surgeons traditionally aim to make the anastomosis as large as possible, even extending the incision into branching pulmonary veins [1, 7, 8]. However, determining the optimal size of the anastomosis preoperatively remains unclear.

One factor that influences the surgical outcome is the size of the vessels, which, according to the continuity equation, has a significant impact on blood flow velocity. The continuity equation describes how an increase in the size of a vessel reduces the velocity of blood flow, affecting the hemodynamics at the surgical site. This relationship is crucial, as it implies that both the size of the anastomosis and the vessel must be carefully considered to achieve optimal flow conditions and reduce the risk of PVO.

In clinical practice, the weight of TAPVC patients is directly related to their blood volume, meaning heavier patients require larger anastomoses to accommodate the increased blood flow. However, many TAPVC cases involve thin and small venous confluences, limiting the ability to create a wide incision, which further complicates surgical planning. To date, no study has provided a method for preoperatively predicting the size of the anastomosis needed for TAPVC patients, and the difficulty of building animal models for TAPVC adds to the challenge. The high risk and complexity of reoperations make it ethically challenging to design prospective research on this issue, emphasizing the need for reliable preoperative planning tools.

Computational fluid dynamics (CFD) has proven to be a powerful tool in hemodynamic research, enabling the creation of numerical models of the cardiovascular system. CFD has been used extensively to study both acquired heart diseases [9–11] and congenital heart diseases [12–14]. In our previous study [15], we developed a patient-specific CFD model of TAPVC, which coupled the simulation of blood flow with vessel interactions at the anastomosis, known as a fluid–structure interaction (FSI) model.

In this study, we extended our previous work by using the FSI model based on more patient-specific imaging data. It is well known that larger anastomoses generally yield better surgical results, however our study also considers the impact of vessel size, as informed by the continuity equation. We designed a series of incremental anastomosis sizes for specific patients and analyzed the relationships between the calculated results, patient weight, and pulmonary venous size. From these relationships, we derived a mathematical equation to calculate the sufficient anastomosis size for TAPVC patients, which we believe holds great potential to guide surgical treatment in clinical practice.

## Methods

### Clinical Data and Geometry Building

This work is retrospective research that employs historical medical imaging data from patients without any identification. In this study, we used clinical data from seven children in our hospital who were diagnosed with supra-cardiac and infra-cardiac TAPVC. Supra-cardiac type 5 cases and infra-cardiac 2 cases. The supra-cardiac and infra-cardiac type respectively account for 30–40% and 20–30% of all TAVPC cases [1].

**Fig. 1:**
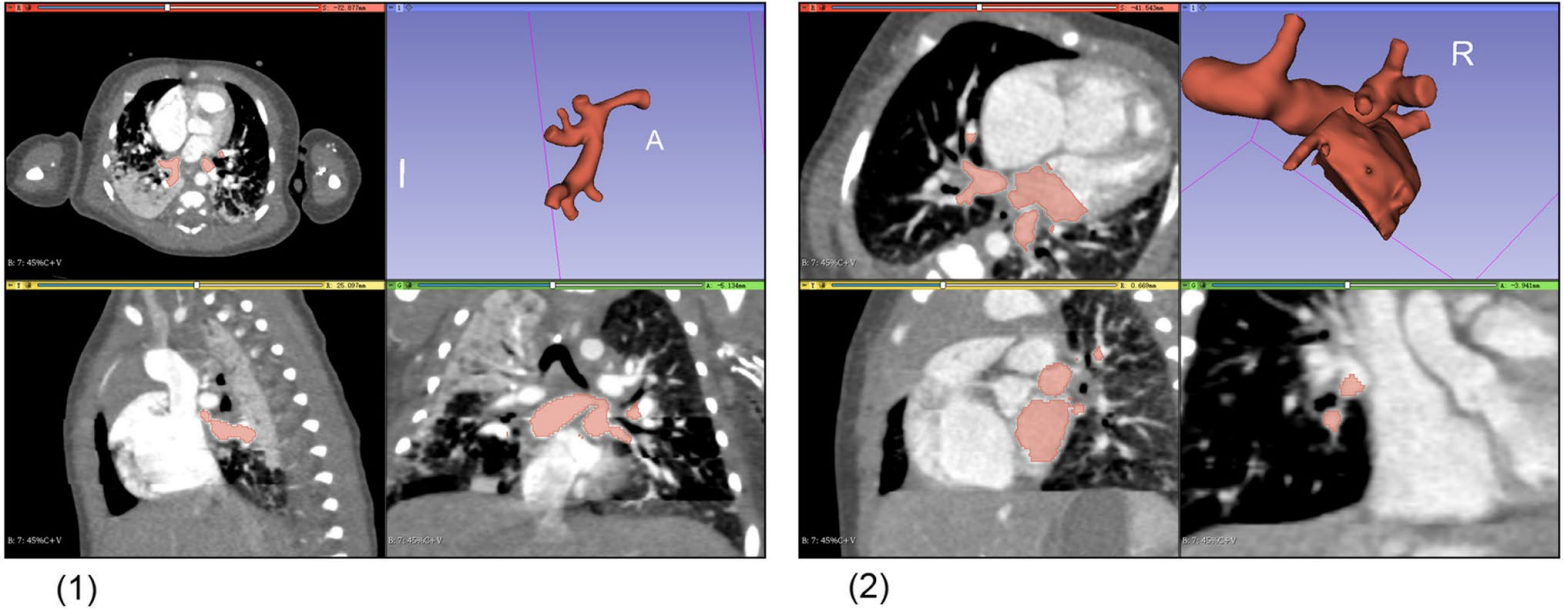
Original data and acquired 3D geometry for two cases, including two components: left atrium and pulmonary veins. (1) Original data and 3D geometry for Case C; (2) original data and 3D geometry for Case D

The original cross-sectional computer tomography angiography (CTA) images were acquired by 64-slice multidetector computer tomography (CT) and used for three-dimensional reconstruction with the open-source software 3D Slicer 4.10. We reconstructed the geometries of pulmonary veins and venous confluence, as shown in Fig. 1.

**Fig. 2:**
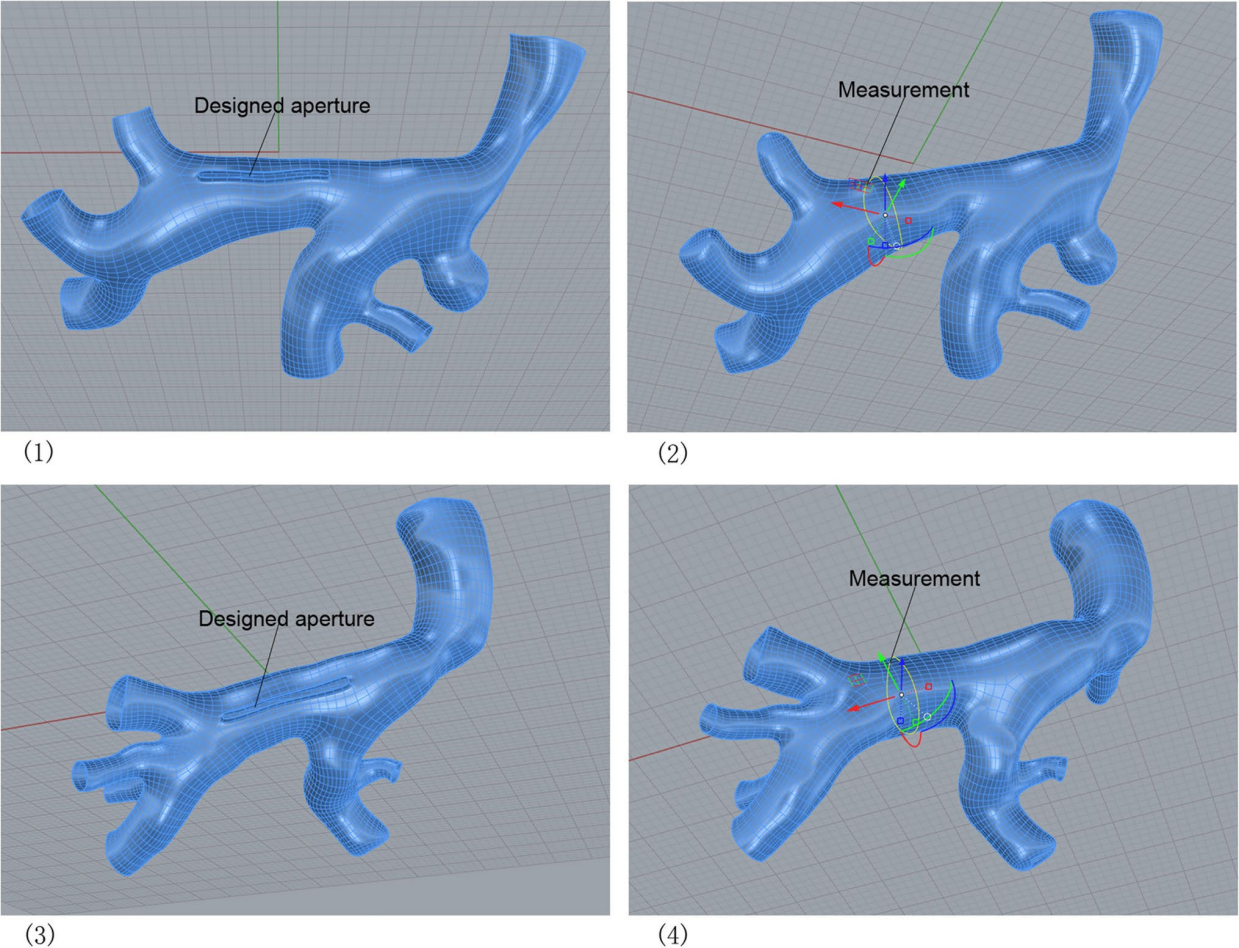
Rebuild the geometry for two cases and measure the circumference of the vessel: make an aperture on the confluence of pulmonary veins, and the aperture protrudes toward the left atrium at the proper location for suturing. (1) Rebuilt geometry for Case C; (2) Measurement approach for vascular circumference of Case C; (3) Rebuilt geometry for Case D; (4) Measurement approach for vascular circumference of Case D

The 3D model of the pulmonary vein was reconstructed based on a CT scan, and an aperture with a 0.5 mm high rim was designed along the vein. The aperture protruded toward the left atrium and is shown in Fig. 2.

This aperture simulates anastomosis, the transition part between pulmonary veins and left atrium after surgery, which designed to be 0.5 mm high. We set six sized lengths of aperture for each case. These apertures also simulated the incisions made by the surgeon before suturing. We generated the aperture and measured it using the commercial software Rhino 3D 7.1. We also measured the size of venous confluence at the part where there was no interference by connected venous branches in Rhino 3D. The measurement is also shown in Fig. 2.

In the FSI model, the simulation requires two independent entities, vessel and blood entities. Both entities included protruding anastomosis regions.

The vessel with anastomosis body reconstruction was made by using SpaceClaim and DesignModeler software (ANSYS, 2021R2, Academic). The geometries of both entities were meshed separately: one entity represented the fluid-blood part, and the other entity represented the structure-vessel part. We defined the wall thickness as approximately 10% of the vessel diameter [15].

### Computational simulation and calculation

The geometrically shaped inlets were defined as the pulmonary venous branches. The outlet was defined as the opening of the anastomosis. The input data included parameters given by the mass flow inlet and pressure outlet values.

The value of inflow mass is derived from the necessary cardiac output of the infant’s left ventricle and is calculated in accordance with body weight [16]. The model outlet was set as pressure-outflow and corresponded to the left atrial pressure, with values ranging from 4–12 mmHg for typical infants [17]. Left atrial pressure varies over time with the heart cycle, and we set a time-varying pressure for the outlet based on our previous study [15]. The results would also vary over time with the heart cycle; we can obtain the peak velocity of blood flow. The outer surface of the anastomosis in the fluid part was set as the system coupling region. In the vessel part, the inner surface of the anastomosis on the vessel was set as the fluid‒solid interface.

The Fluent package (ANSYS, 2021R2, Academic) was employed to solve Navier‒Stokes equations on the fluid (blood) body. The structure (vessel) body was calculated in the Transient Structural package (ANSYS, 2021R2, Academic). Two-way coupling of fluid and structure coupling was performed in the System Coupling package (ANSYS, 2021R2, Academic).

The details of the computational methods have been previously described [15]. The material of the vessel was assumed to be an isotropic elastic material in transient structure. We set a uniform Poisson's ratio of 0.48 and Young’s modulus of 0.5 MPa for vessels. These values were adopted from literature precedent rather than patient-specific measurements, consistent with our previous work [15] The results of the calculations include the fluid part and structural part. The peak velocity of fluid at the outlet is the main evaluations.

The coupled FSI solver employed in this study (ANSYS Fluent + Transient Structural) has been validated by the ANSYS Inc. development team itself through multiple benchmark cases and experimental comparisons [18], confirming the accuracy, reliability and numerical stability of the solver framework.

To ensure the numerical accuracy and stability of the simulation results, mesh and time-step independence analyses were performed on two representative patient-specific models (one supra-cardiac and one infra-cardiac). Detailed quantitative results and mesh visualizations are provided in the Supplementary Material.

### Data analysis and equation generation

The velocity of blood flow passing through the anastomosis is a critical indicator of whether the anastomosis is stenotic [2, 3]. After calculating the blood flow velocity at the simulated anastomosis, we investigated its relationship with patient-specific parameters, including body weight and pulmonary venous circumference. Statistical analyses were performed using MATLAB (R2024b, MathWorks Inc., Academic License). Data normality was assessed, confirming that the data followed a normal distribution. Subsequently, Pearson’s correlation coefficient was calculated to evaluate the linear relationship between blood flow velocity and M/LC, and a simple linear regression analysis was performed to model this relationship. The coefficient of determination (*R*^2^) was computed to assess the goodness-of-fit of the regression model. A significance threshold of *p* < 0.05 was set for all analyses. The MATLAB scripts used to perform these analyses are provided in the supplementary materials.

It is well known that the velocity of blood flow is positively correlated with the flow rate, which in turn is directly related to the patient’s body weight.

Summarizing the all proven and observed correlations, we define the following relations:

1. When considering the influence of size of PVC:

1 $$V\propto \frac{M}{L*C}$$

where *V* is the peak velocity of blood flow, *M* is the body weight of the patient, *L* is the length of the simulated anastomosis, and *C* is the circumference of the PVC. This relation suggests that for a given body weight, increasing the anastomotic length or the circumference of the venous confluence will reduce the velocity of blood flow, highlighting the significance of these dimensions in results.

2. Without considering the size of the PVC:

2 $$V\propto \frac{M}{L}$$

In this relation, *V* remains the maximal velocity of blood flow, *M* is the patient’s body weight, and *L* is the length of the anastomosis. This relation focuses solely on the anastomosis size, disregarding the vessel dimensions. It suggests that, when the vessel size is not considered, the blood flow velocity is determined primarily by the patient’s weight and the length of the anastomosis.

For specific patient, the body weight and size of PVC are unique, making the $\frac{M}{C}$ a constant for that individual.

### Validation

To validate the proposed equations, we analyzed 22 cases of total anomalous pulmonary venous connection, all of which were either supra-cardiac or infra-cardiac types and had previously undergone surgical treatment at our institution. Each case included surgical records of the anastomosis length at the pulmonary vein confluence, measured intraoperatively, and preoperative CTA examinations, which allowed us to measure the circumference of the PVC through three-dimensional reconstruction. All cases underwent direct suturing of the PVC to the left atrium, and those treated with ‘sutureless’ techniques were excluded from the analysis.

Our validation focused on the relationship defined in Eq. (1), which incorporates the size of the PVC. For each case, we input the relevant patient-specific parameters into the equation and calculated the corresponding anastomotic flow velocities. We then compared the calculated velocities with the actual peak velocities measured via postoperative echocardiography.

The difference between the calculated and measured velocities was quantified as the mean absolute error (MAE), with a 95% confidence interval (CI) for the mean difference calculated across all 22 cases. To further evaluate the agreement between the calculated and measured velocities, we conducted a Bland–Altman analysis. A paired t-test was conducted to assess whether there was a statistically significant difference between the two sets of velocities, with a significance threshold set at *p* < 0.05. The MATLAB scripts used to perform these analyses are provided in the supplementary materials.

### Results

The hemodynamic parameters of blood and magnitude of pulmonary veins, such as flow velocity, length of anastomosis domain and circumference of PVC, were obtained.

**Fig. 3:**
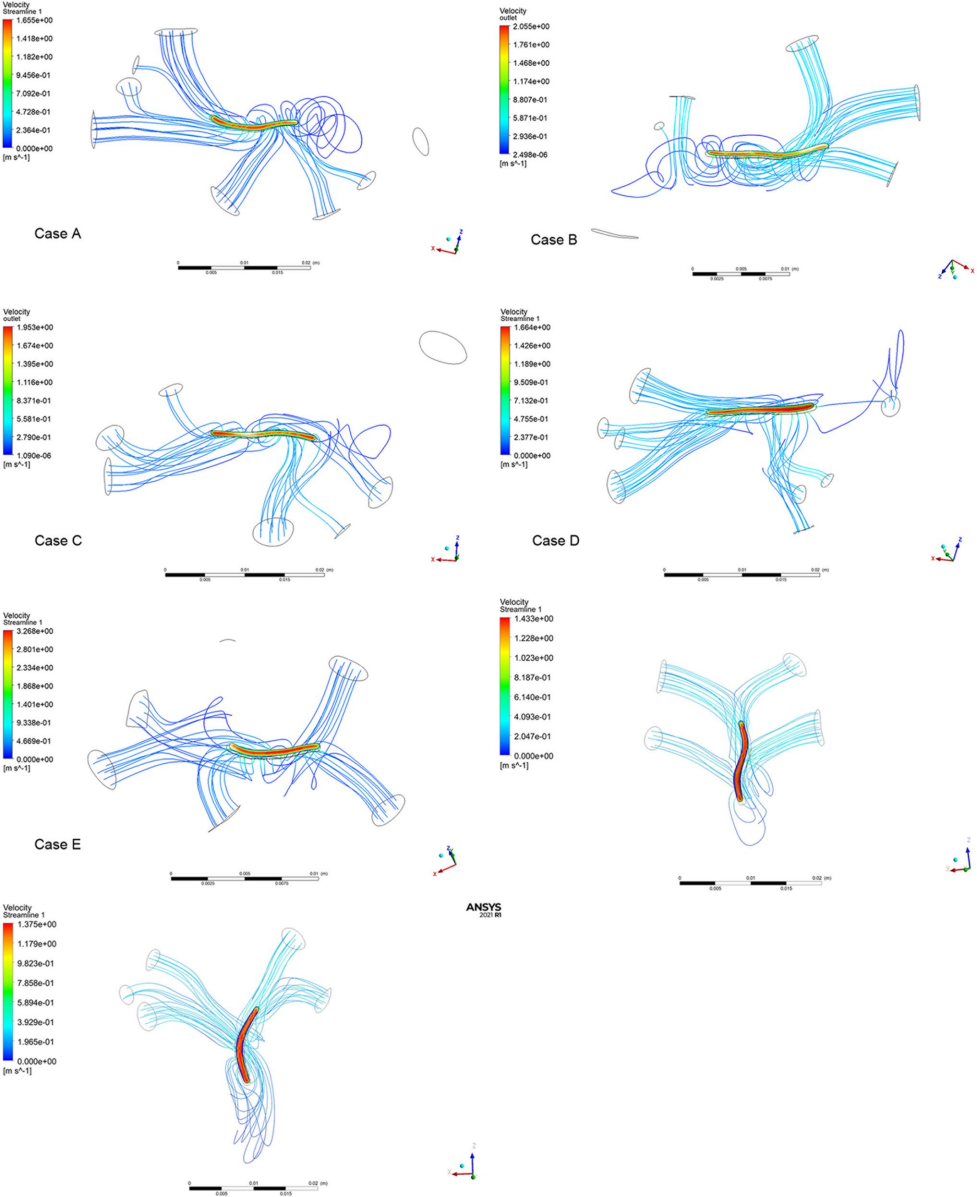
Streamline of blood flow velocity through the anastomosis for five different TAPVC cases (A-G)

**Fig. 4:**
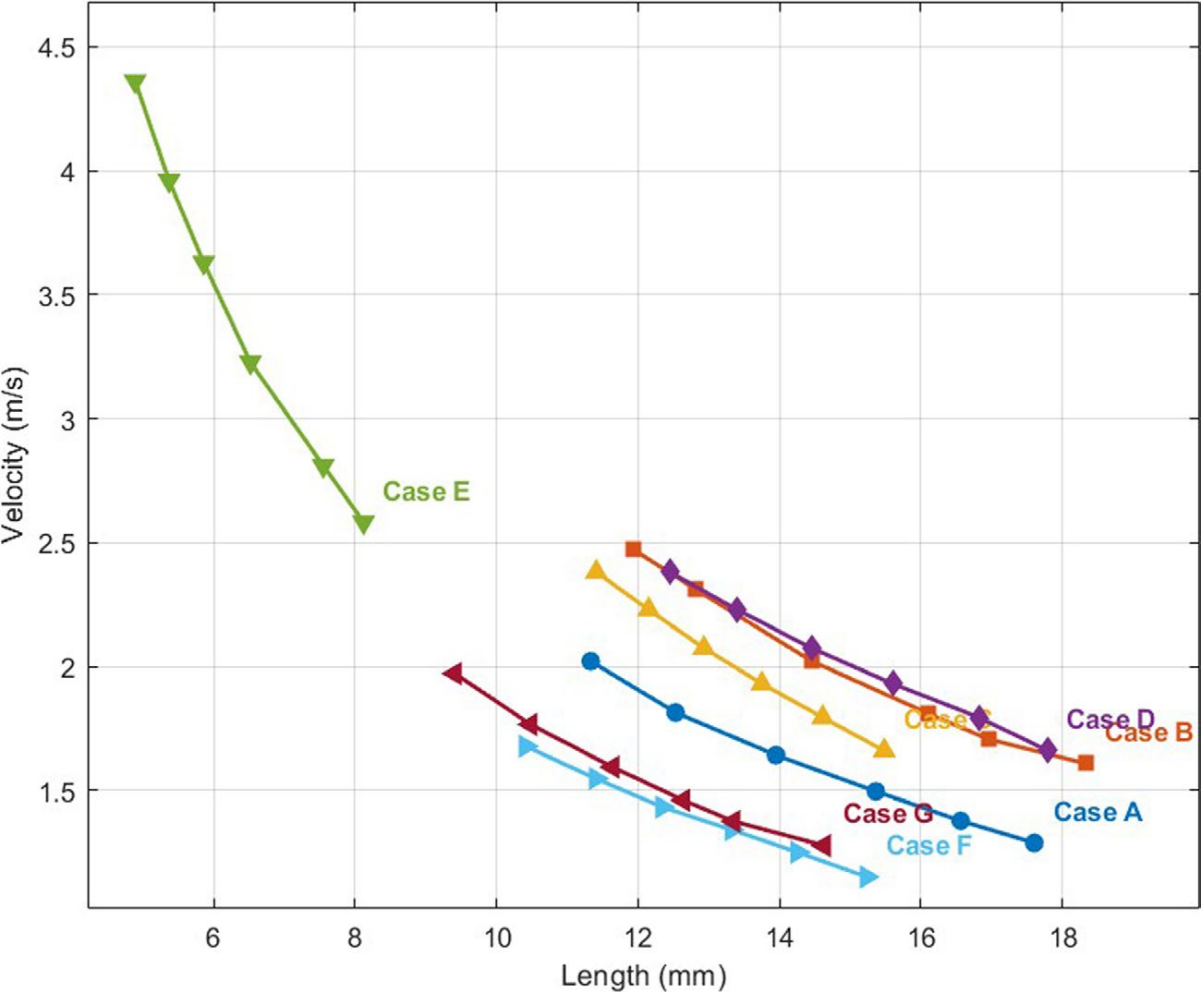
The calculated velocity result of each case and the relations between the velocity and the length of anastomosis of these cases

For each of the seven cases, one peak velocity of blood flow through the anastomosis domain was selected and illustrated in Fig. 3. The velocity calculation results of all seven cases with different lengths of anastomosis are summarized in Table 1 and plotted in Fig. 4. We found that the velocity of blood flow was obviously negatively correlated with the length of the simulative anastomosis.

**Table 1:** Calculated data for each case

Case A: Supra-cardiac **M** = 4.06 kg **C** = 28.6 mm						
**L**(mm)	11.33	12.53	13.53	15.36	16.56	17.60
**Velocity** (m/s)	2.021	1.814	1.642	1.496	1.377	1.288
**M/LC**	0.01253	0.01133	0.00982	0.00924	0.00857	0.00815
**M/L**	0.3583	0.3240	0.2910	0.2643	0.2452	0.2307
Case B: Supra-cardiac** M** = 3.28 kg **C** = 18.5 mm						
**L**(mm)	11.93	12.82	14.45	16.1	16.95	18.33
**Velocity** (m/s)	2.472	2.310	2.022	1.810	1.707	1.608
**M/LC**	0.01486	0.01383	0.01101	0.01010	0.00967	0.00967
**M/L**	0.2749	0.2559	0.2270	0.2037	0.1935	0.1789
Case C: Supra-cardiac **M** = 3.64 kg **C** = 21.8 mm						
**L**(mm)	11.41	12.15	12.93	13.75	14.64	15.48
**Velocity** (m/s)	2.380	2.229	2.074	1.930	1.794	1.660
**M/LC**	0.01461	0.01372	0.01289	0.01212	0.01088	0.01077
**M/L**	0.3190	0.2996	0.2815	0.2647	0.2491	0.2351
Case D: Supra-cardiac **M** = 3.68 kg **C** = 24.2 mm						
**L**(mm)	12.45	13.40	14.45	15.6	16.82	17.79
**Velocity** (m/s)	1.997	1.797	1.636	1.495	1.404	1.317
**M/LC**	0.01221	0.01135	0.01052	0.00914	0.00904	0.00855
**M/L**	0.2956	0.2746	0.2547	0.2359	0.2189	0.2069
Case E: Supra-cardiac **M** = 1.95 kg **C** = 14.9 mm						
**L**(mm)	4.89	5.37	6.13	6.52	7.55	8.12
**Velocity** (m/s)	4.361	3.960	3.629	3.227	2.810	2.582
**M/LC**	0.02685	0.02445	0.02241	0.0204	0.01739	0.01617
**M/L**	0.3990	0.3633	0.3326	0.2991	0.2582	0.2401
Case F: Infra-cardiac **M** = 3.35 kg **C** = 29.61 mm						
**L**(mm)	10.41	11.4	12.34	13.32	14.25	15.23
**Velocity** (m/s)	1.678	1.548	1.433	1.341	1.251	1.151
**M/LC**	0.01087	0.00992	0.00917	0.00849	0.00794	0.00743
**M/L**	0.3218	0.2939	0.2715	0.2515	0.2351	0.2200
Case G: Infra-cardiac **M** = 3.80 kg **C** = 30.83 mm						
**L**(mm)	9.41	10.48	11.64	12.64	13.35	14.63
**Velocity** (m/s)	1.971	1.766	1.594	1.46	1.375	1.277
**M/LC**	0.01310	0.01176	0.01059	0.00975	0.00923	0.00842
**M/L**	0.4038	0.3626	0.3265	0.3006	0.2846	0.2597

**Velocity**, calculated maximal velocity of blood flow; **L**, incremental lengths of anastomosis; **M**, weight of specific patient; **C**, circumference of specific patient’s pulmonary venous confluence; **M/LC**, weight was divided by circumference and lengths of anastomosis; **M/L**, weight was only divided by lengths of anastomosis

**Fig. 5:**
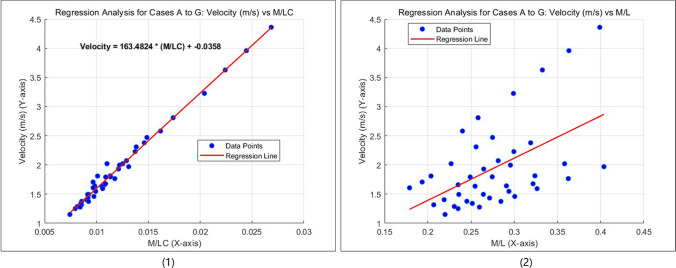
(1) Relationship between blood flow velocity (V) and the ratio of patient weight to anastomosis length and pulmonary venous confluence circumference (M/LC). The data points represent results from all five cases; (2) Relationship between blood flow velocity (V) and the ratio of weight to anastomosis length (M/L), excluding the circumference of the pulmonary venous confluence (C)

The measured circumference of PVC and weight from clinical data of all cases are also listed in Table 1. By relation (1), the weight was divided by circumference and incremental lengths of anastomosis from each case, named variate M/LC, and the results are also listed in Table 1. By relation (2), the weight was divided only by lengths of anastomosis, named variate M/L, are also listed in Table 1. From Table 1, all the calculated velocity data and all the variates M/LC and M/L were extracted and plotted, where the velocity data are the Y axis, as shown in Fig. 5.

A strong positive correlation between velocity and M/LC was observed. The plotted graph, shown in Fig. 5 (1), was generated using MATLAB (R2024b, MathWorks Inc., Academic License). The fitted linear regression curve and its formula are also displayed in Fig. 5 (1). The Pearson correlation coefficient (*R* = 0.9942, *p* < 0.0001) indicates a significant positive linear correlation between velocity and M/LC. The regression model demonstrated a good fit, as evidenced by the coefficient of determination (*R*^2^ = 0.9885).

According to the results of curve fitting and regression analysis and due to the very tiny intercept number, we can generate the following equation:

3 $$V=163.48.\frac{M}{L*C}$$

where *V* is the maximal velocity of blood flow in meters per second; *M* is the body weight of the patient in kilograms; *L* is the length of the simulative anastomosis in millimeters; C is the circumference of PVC in millimeters.

We can also deduce another equation from above:

4 $$L=163.48\frac{M}{V*C}$$

Equation (4) demonstrates that *L*, the length of anastomosis, which is equivalent to the length of incision that is enough to result in the aimed velocity of blood flow, can be determined by this equation.

In contrast, Fig. 5 (2) shows the relationship between velocity (*V*) and the simple ratio *M*/*L*, which excludes the vessel circumference variable. To comprehensively evaluate this relationship, data from all cases were combined for regression analysis. The results indicated a moderate positive correlation between *M*/*L* and V (*R* = 0.5272, *p* < 0.05), while the coefficient of determination (*R*^2^ = 0.2779) suggested that this relationship explains only 27.79% of the variation in *V*. This finding highlights the critical role of incorporating circumference (*C*) in predicting flow velocity and optimizing anastomosis design. Without considering size of vessel, the relationship between *M*/*L* and *V* remains inconsistent across patients, limiting the model's applicability to clinical decision-making.

For validation, the clinical data, which was echocardiographic measured, and the results calculated using Eq. (3) are presented in Table 2. The mean absolute error (MAE) between the calculated and measured velocities was 0.1742. The paired t-test was performed to assess the statistical difference between the calculated and measured velocities, yielding a *p*-value of 0.1247 and a 95% confidence interval of [− 0.0230, 0.1765]. These results indicate that there is no statistically significant difference between the two groups.

**Table 2:** Clinical data and calculation for validation

Case	Weight(kg)	Length of incisions (mm)	Circumference (mm)	Type of TAPVC	Calculated velocity (m/s)	Echo measured velocity (m/s)
01	3.27	18	19.2	infra-cardiac	1.547	1.66
02	3.50	20	25.7	infra-cardiac	1.113	1.27
03	3.65	20	21.8	supra-cardiac	1.368	1.52
04	4.80	20	28.1	supra-cardiac	1.396	1.60
05	2.56	15	22.9	infra-cardiac	1.218	1.32
06	2.58	15	21.9	supra-cardiac	1.260	1.25
07	3.30	12	24.9	supra-cardiac	1.805	1.30
08	3.80	20	19.2	supra-cardiac	1.618	1.40
09	3.72	18	18.4	supra-cardiac	1.836	1.64
10	2.90	16	16.6	supra-cardiac	1.785	1.23
11	2.86	15	25.2	infra-cardiac	1.237	1.37
12	3.05	18	23.7	supra-cardiac	1.169	1.30
13	3.86	15	21.5	supra-cardiac	1.957	1.52
14	4.06	16	28.6	supra-cardiac*	1.450	1.53
15	3.68	18	24.2	supra-cardiac*	1.381	1.35
16	3.28	15	18.5	supra-cardiac*	1.932	1.88
17	3.64	16	22.5	supra-cardiac	1.653	1.26
18	3.35	15	29.6	infra-cardiac*	1.233	1.21
19	3.80	15	30.8	infra-cardiac*	1.345	1.28
20	4.26	18	27.6	supra-cardiac	1.402	1.25
21	3.40	15	26.3	supra-cardiac	1.409	1.40
22	3.26	15	24.6	supra-cardiac	1.444	1.33

^*^ Case was used in CFD simulation in this study

To further evaluate the agreement between the calculated and measured velocities, we conducted a Bland–Altman analysis. This analysis revealed a mean difference of 0.0767, with the standard deviation of 0.2250 and with the differences distributed around the mean within limits of agreement (− 0.3643 to 0.5178).

### Discussion

The size of the anastomosis is critical in TAPVC surgery, with current practices favoring the largest possible anastomosis to ensure adequate blood flow from the pulmonary veins to the left atrium in both supra-cardiac and infra-cardiac TAPVC patients [1, 7, 8]. However, the optimal anastomosis size for individual patients remains unclear. Cheng et al. proposed a numerical model using a rigid pulmonary wall to simulate the relative cross-sectional area (RCSA) based on body surface area [19], but variations in RCSA for different geometries were not explored. Our findings highlight the key role of PVC in predicting surgical outcomes. Studies on arteriovenous fistula models [20, 21] have shown that a larger anastomosis reduces pressure drop and increases proximal arterial inflow. Some great studies have investigated risk factors for postoperative PVO [3, 5, 22], these mainly focused on clinical data, such as age, weight, arrest time, and TAPVC type, rather than pulmonary vein or anastomosis size. Our study demonstrates the significant impact of these anatomical factors on simulated outcomes.

Our study revealed that when the PVC circumference is considered as a variable, the correlation between predicted flow velocities and actual clinical outcomes becomes significantly stronger. This indicates that the confluence size is a critical factor that should not be overlooked in preoperative planning.

In contrast, models that excluded the confluence circumference exhibited a weaker correlation when comparing results across different cases. This finding underscores the limitations of relying solely on anastomosis size and patient weight without considering the size of PVC. Consequently, our findings advocate for the integration of this key anatomical parameter into surgical planning to optimize outcomes and minimize the risk of postoperative complications such as PVO. From a clinical perspective, postoperative velocity is already widely used as an indicator of anastomotic patency, although the specific thresholds vary among studies [5, 6]. Accordingly, we interpret velocity as a continuous parameter, where lower values generally suggest reduced risk of obstruction. Our predictive model therefore provides a quantitative tool to help surgeons preoperatively target lower velocities by considering not only patient's weight but also confluence circumference.

In addition to the role of anastomosis length and confluence size, the vessel wall properties also affect the hemodynamic environment. In our simulations, the wall material was assumed isotropic with uniform elastic parameters, while wall thickness was scaled according to patient body weight [15]. As a result, although the same elastic parameters were applied, the effective elasticity varied across cases, partially preserving inter-patient biomechanical differences. Future studies including sensitivity analyses of vascular elasticity parameters will help to further validate the robustness of this approach.

The equation derived from our study, incorporating real clinical data, was validated by comparing the calculated flow velocities with those measured via echocardiography post-surgery. The results demonstrated no significant difference, thereby validating the model’s accuracy. However, the number of clinical cases used for validation was relatively small, and there are limitations to consider. For example, the anastomosis size data were obtained using calipers during surgery, which may introduce variability in measurements due to differences in the accuracy of the instruments used. Moreover, these measurements were taken while the heart was arrested and the pulmonary veins were relaxed, whereas our model simulates hemodynamics during postoperative conditions, when the veins are under tension. This discrepancy could partially account for any differences observed between the calculated and clinical results. However, because our predictive equation is linear, such differences translate proportionally into velocity without altering the structure or validity of the equation. We note that physiologic conditions may cause modest variations in input values, but the overall predictive relationship remains robust.

Additionally, some cases lacked complete intraoperative records of the anastomosis dimensions, making them unsuitable for validation analysis. For instance, among the seven cases used for CFD simulations, one lacked clinical documentation of the anastomosis size and was therefore excluded from the validation process. This highlights the need for more comprehensive and standardized data collection in future studies to improve the robustness of validation efforts.

Another one of the five cases used for CFD simulations was also excluded from validation due to the exceptionally small PVC, which underwent the sutureless technique during surgery. This approach avoids direct anastomosis and is incompatible with our model. While the sutureless technique reduces anastomotic stenosis, it increases risks of bleeding and thrombus formation due to vascular intima exposure [23, 24].

In summary, while the size of the anastomosis remains a crucial factor in TAPVC surgery, our study highlights the essential role of the pulmonary venous confluence circumference in refining predictive models and improving surgical outcomes. Surgeons should consider this variable in preoperative planning to ensure more accurate predictions of flow dynamics and better patient outcomes.

### Limitations

This study has several limitations. First, the sample size remains small and is derived from a single institution, which may limit the generalizability of the findings. Although both supra-cardiac and infra-cardiac cases were included, the overall cohort is still limited, and external validation in larger, multicenter cohorts will be essential to confirm the robustness and broader applicability of the model. Second, vessel wall elasticity parameters were adopted from literature precedent rather than measured individually in patient-specific vessels. Third, long-term clinical outcomes such as postoperative PVO incidence, reintervention, and survival were not available for correlation with the computational predictions. Finally, while the model demonstrated very high correlation coefficients with clinical data, this performance in a relatively small dataset may reflect potential overfitting, underscoring the need for larger external validation.

## Supplementary Information

Below is the link to the electronic supplementary material.

Supplementary file1 (DOCX 416 KB)

Supplementary file2 (M 2 KB)

Supplementary file3 (M 2 KB)

Supplementary file4 (M 2 KB)

## Data Availability

The datasets generated and analysed during the current study are available from the corresponding author on reasonable request.

## Glossary Abbreviations

TAPVC: Total anomalous pulmonary venous connection
PVO: Pulmonary venous obstruction
PVC: Pulmonary venous confluence
CFD: Computational fluid dynamics
FSI: Fluid-structure interaction
CTA: Computer tomography angiography
CT: Computer tomography
RCSA: Relative cross-sectional area
