# Supplementary material for: Computational Predictive Modeling of Surgical Outcomes in Total Anomalous Pulmonary Venous Connection: Assessing the Impact of Pulmonary Venous Confluence Size on Preoperative Planning
Source: J Cardiovasc Transl Res. 2026 Feb 4;19(1):7. doi: 10.1007/s12265-025-10725-9 (PMC12872693; doi:10.1007/s12265-025-10725-9)
Supplement: Supplementary file 1 — Supplementary file1 (DOCX 416 KB) [file 12265_2025_10725_MOESM1_ESM.docx]

Mesh and Time-Step Independence Study

**1. Mesh Independence Study**

To ensure mesh-independent results, two representative models were selected: one supra-cardiac (Case B) and one infra-cardiac (Case F). Each model was simulated using three different mesh densities:

1. Original (as used in the main manuscript)
2. Medium refinement
3. High refinement

Both fluid and solid domains were refined accordingly. The table below summarizes the mesh configurations, peak velocities, and computational cost. Differences in peak velocity compared to the original mesh are also presented. The **fluid body meshes** are visualized below (Figure S1) to demonstrate the variation in mesh resolution for clarity. The **solid domain meshes** followed the same refinement strategy but are not shown here.

Table S1: Mesh density independence analysis using two representative cases

| Case | Mesh Density | Grid Size (fluid/solid) | Peak Velocity (m/s) | Δ vs Original (%) | CPU Time(s) |
| --- | --- | --- | --- | --- | --- |
| B1 | Original (in Paper) | 2.089mm/0.20mm | 2.022 | - | 2.1482E+03 |
| B2 | Medium Refinement | 0.5mm/0.15mm | 2.023 | +0.05% | 3.4246E+03 |
| B3 | High Refinement | 0.3mm/0.10mm | 2.026 | +0.20% | 7.1615E+03 |
| F1 | Original (in Paper) | 2.220mm/0.44mm | 1.433 | - | 9.8809E+02 |
| F2 | Medium Refinement | 0.5mm/0.15mm | 1.431 | -0.14% | 4.2251E+03 |
| F3 | High Refinement | 0.3mm/0.1mm | 1.429 | -0.28% | 9.3335E+03 |

Case B: one case of supra-cardiac cases; Case F: one case of infra-cardiac cases


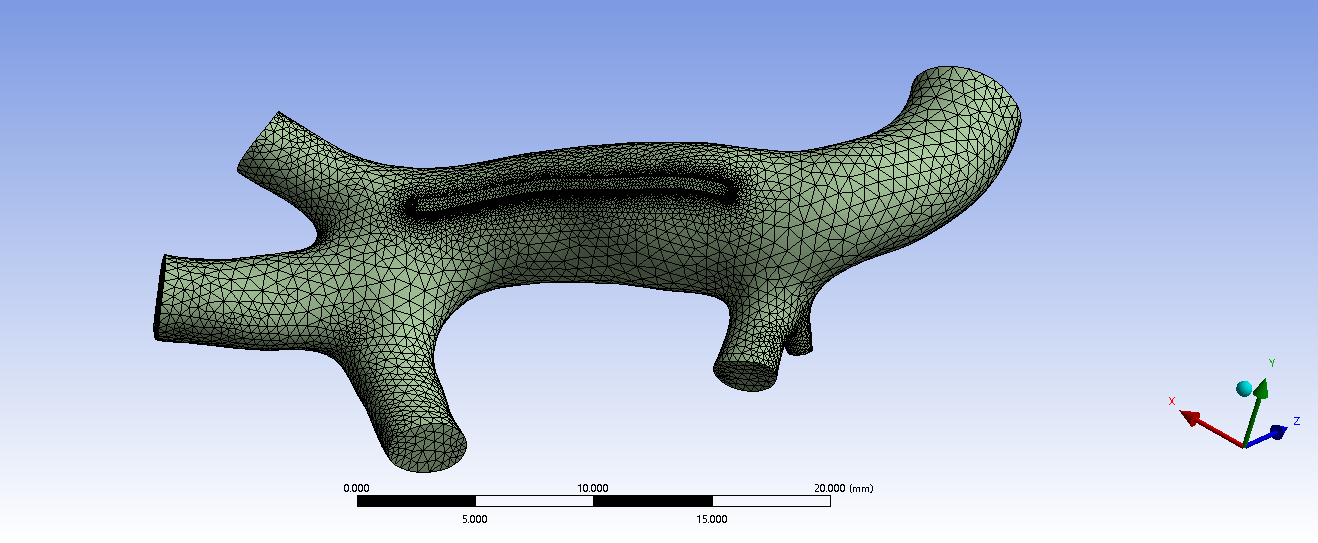


Figure S1.1: Low mesh density (original) of case B Fluid domain


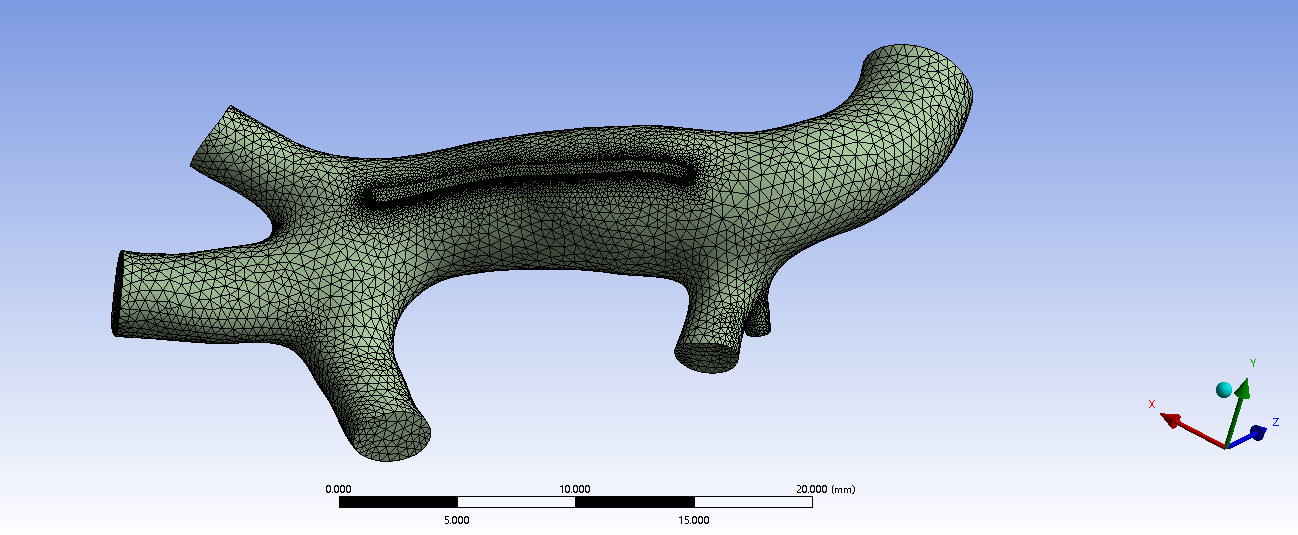


Figure S1.2: Medium refinement mesh


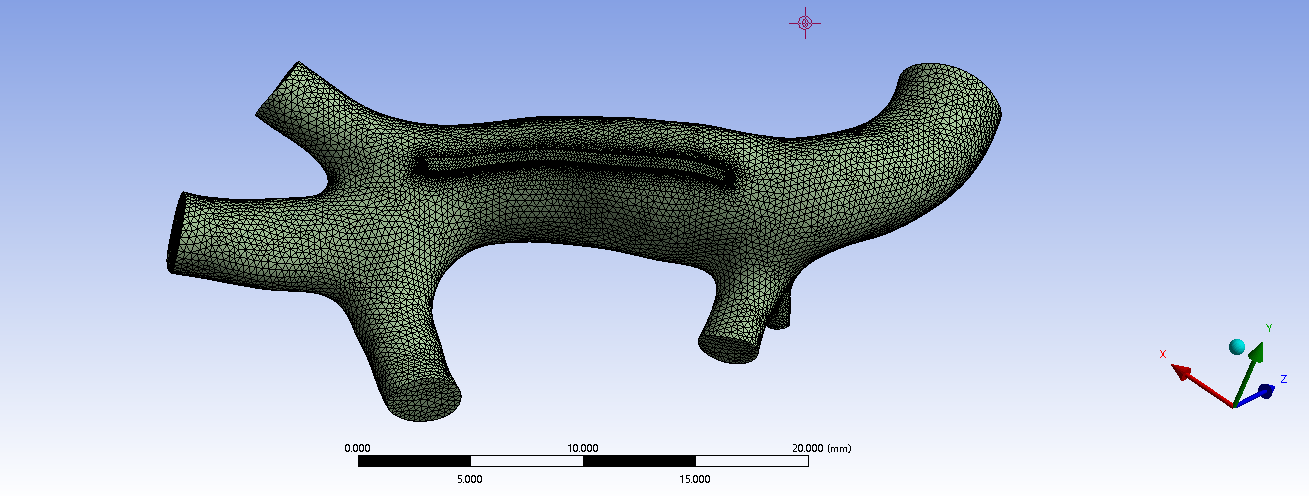


Figure S1.3: High refinement mesh

| Case |  | Time Step (s) | Peak Velocity (m/s) | Δ vs Original (%) | CPU Time(s) |
| --- | --- | --- | --- | --- | --- |
| B1 | T (in Paper) | 0.01 | 2.022 | - | 2.1482E+03 |
| B2 | T/2 | 0.005 | 2.022 | 0.00% | 4.1649E+03 |
| B3 | T/4 | 0.0025 | 2.023 | +0.05% | 8.2803E+03 |
| F1 | T (in Paper) | 0.01 | 1.433 | - | 9.8809E+02 |
| F2 | T/2 | 0.005 | 1.433 | 0.00% | 1.8037E+03 |
| F3 | T/4 | 0.0025 | 1.433 | 0.00% | 3.7014E+03 |

**2. Time-Step Independence Study**

To assess time-step sensitivity, the same two representative models (Case B and Case F) were simulated using three level of time steps:

1. T (used in the main manuscript, 0.01 s)
2. T/2 (0.005 s)
3. T/4 (0.0025 s)

The total simulation time was fixed at 1 seconds for all simulations. The resulting peak velocities and CPU time consumption are shown below.

Table S2: Time-step independence analysis using two representative cases

Case B: one case of supra-cardiac cases; Case F: one case of infra-cardiac cases
